# Safety and Early Clinical Outcomes Following Repair of Very Large Hiatus Hernia in Octogenarians

**DOI:** 10.1002/wjs.12569

**Published:** 2025-04-13

**Authors:** Mathew A. Amprayil, Tanya Irvine, Sarah K. Thompson, Tim Bright, David I. Watson

**Affiliations:** ^1^ Flinders University Discipline of Surgery Flinders Medical Centre Bedford Park Australia

**Keywords:** esophagus, hernia, hiatus, octogenarian

## Abstract

**Background:**

Very large hiatus hernias are often symptomatic, impact quality of life, and are increasingly encountered in aging populations. Laparoscopic repair offers excellent clinical outcomes. However, surgeons can be reluctant to offer surgery to the elderly due to concerns about morbidity and mortality. To determine safety, we evaluated outcomes following repair of very large hiatus hernias in patients aged 80 years and older and compared them to younger patients.

**Methods:**

Data were extracted from a prospective database. Patients who underwent operative repair of a very large hiatus hernia (> 50% intrathoracic stomach) between 2000 and 2023 were included and categorized into groups based on age: young (< 70 years), older (70–79 years), and octogenarian (≥ 80 years). Perioperative and early postoperative clinical outcomes were determined and compared.

**Results:**

1353 patients underwent surgery (< 70 years: 733 [54.2%], 70–79 years: 451 [33.3%], and ≥ 80 years: 169 [12.5%]). Rates of total intrathoracic stomach were commonest in octogenarians (11.6% vs. 20.4% vs. 32.5% and *p* < 0.001). Young and older patients were more likely to undergo elective surgery for heartburn (56.6% vs. 44.4% vs. 29.0% and *p* < 0.001), whereas octogenarians more likely underwent emergency surgery for gastric volvulus (5.4% vs. 6.6% vs. 14.5% and *p* = 0.019). Conversion to open surgery (1.1% vs. 1.1% vs. 5.0% and *p* = 0.002) and length of stay (2.69 vs. 3.19 vs. 4.62 days and *p* < 0.001) were greater in the octogenarian group. Major complications (4.2% vs. 5.1% vs. 8.1% and *p* = 0.120) and return to theater rates (2.6% vs. 2.9% vs. 2.7% and *p* = 0.925) were similar. Thirty‐day mortality rates were low for all groups but highest in octogenarians (0.3% vs. 0.4% vs. 1.8% and *p* = 0.048). Adverse outcomes were more likely with emergency presentations, which were more common in octogenarians.

**Conclusion:**

Despite a higher rate of emergency surgery in octogenarians—major complications and overall mortality rates are still acceptably low. Repair of very large symptomatic hiatus hernia should not be withheld from patients aged over 80 who are otherwise fit.

## Graphical abstract

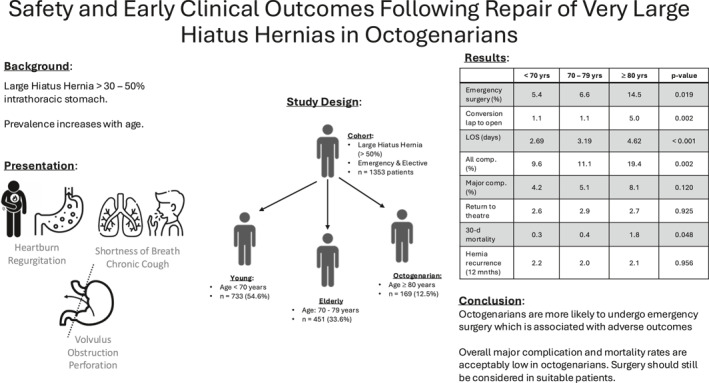

## 1 Introduction

A large hiatus hernia occurs when more than 30%–50% of the stomach lies within the thoracic cavity [1]. They account for 5%–10% of all hiatus hernias [2]. Large hiatus hernias are a significant source of morbidity and negatively impact quality of life [3, 4]. Laparoscopic repair of large hiatus hernias is the standard treatment for symptomatic patients [5]. However, surgeons can be reluctant to offer surgery to elderly patients due to concerns about perioperative morbidity and mortality.

As Western countries age, the octogenarian demographic is increasing. In Australia, octogenarians (patients aged 80 years or greater) are expected to increase from 4% in 2019 to 7.6% in 2066 [6]. As the incidence of large hiatus hernias increases with age, surgeons require a greater understanding of the risk versus benefit profile of surgery in patients aged 80 years or older.

Previous studies have assessed the safety of paraesophageal hiatus hernia repair in octogenarians but fail to mention the size of the hernia [7, 8, 9, 10, 11, 12, 13]. There is an additional level of complexity and morbidity associated with surgical repair of large hiatus hernias, with surgery requiring greater thoracic dissection to reduce the stomach back down into the abdominal cavity. Furthermore, the more widened hiatal defect puts the repair under additional tension. *Cocco et al* have demonstrated that a higher percentage of intrathoracic stomach is predictive of increased operative and postoperative morbidity and persistent reflux symptoms [14].

The literature for “large hiatus hernia” repair in octogenarians is limited to relatively small cohorts. Octogenarians in previous reports were more likely to undergo emergency surgery, had a longer length of stay, and suffered more minor complications. Major complication and mortality rates vary [15, 16, 17, 18, 19]. In this study, we assessed a large cohort who underwent surgery for hernias containing at least 50% of the stomach to determine the safety and perioperative and early clinical outcomes for repair of very large hiatus hernias in octogenarians. Outcomes were compared to younger cohorts.

## 2 Methods

A prospectively collected multicentre audit database was used to identify patients who underwent large hiatus hernia repair between 2000 and 2023 at Flinders Medical Center, Royal Adelaide Hospital, and associated private hospitals in Adelaide, South Australia. Patients were included in this study if they had a very large hiatus hernia identified at preoperative work‐up with endoscopy or imaging and then confirmed at surgery. A very large hiatus hernia was defined as ≥ 50% intrathoracic stomach. The primary indication for surgery was one or more of symptomatic gastroesophageal reflux disease, a hiatus hernia with mechanical symptoms, anemia secondary to Cameron's ulcers, or emergency presentation with gastric volvulus requiring decompression and urgent surgery. Otherwise, fit patients under the age of 70 with asymptomatic very large hiatus hernias were also offered elective repair. Patients undergoing surgery before 2000 were excluded to ensure any learning curve bias was avoided.

Preoperative endoscopy was undertaken in the majority of (but not all) patients. Hernia size was also commonly assessed before surgery using contrast radiology (CT scan or contrast swallow X‐ray). Only patients with more than 50% of the stomach within the hiatus hernia were included in this study. Esophageal manometry and pH monitoring were performed at surgeon discretion, largely in patients undergoing surgery for whom gastroesophageal reflux was the main presentation.

A standard laparoscopic repair was undertaken, and this has been described in detail elsewhere [20]. The surgical steps included: complete hiatal sac dissection, reduction of the sac, intrathoracic stomach and the distal 2–3 cm of esophageal back into the abdomen, closure of the widened hiatus with posteriorly places sutures which were supplemented by anterior sutures when required, and then an anterior partial fundoplication for reflux control and to anchor the stomach below the diaphragm. Hiatal reinforcement with mesh was undertaken in a minority of patients, often within the context of a randomized trial [21]. On the day following surgery, a contrast swallow X‐ray was usually performed to check the integrity of the hiatal repair. Patients were then discharged the next day, but if there were any concerns with the radiology appearances, laparoscopic reexploration was undertaken. Later investigations were only performed in symptomatic patients.

Patients completed a structured symptom questionnaire before surgery, at 3, 6, and 12 months postoperatively, and then yearly after that. The questionnaire included visual analog scales (0–10) to assess heartburn and regurgitation severity, satisfaction scores (0–10), and if they considered their decision to undergo surgery to be correct (yes/no).

To assess the success of surgery, we evaluated short term follow‐up outcomes. This was done by comparing clinical data from the preoperative and 12 months postoperative time points. To maximize the completeness of early follow‐up, if 12‐month data were missing but 2‐year postoperative data were available, then this was substituted. If 2‐year data were also missing, then 6‐month followed by 3‐month data were substituted in that order of priority.

Patients were categorized into three groups based on age for comparison: young (< 70 years), older (70–79 years), and octogenarian (≥ 80 years). The outcomes analyzed included: operative time, length of stay in hospital, all and major complications (Clavien–Dindo ≥ 3a), early reoperation within 30 days of hiatus hernia repair, 30‐ and 90‐day mortality rates, visual analog scores for heartburn and regurgitation, patient satisfaction scores, recurrent hiatus hernias, and reoperations within the first 12 months after surgery.

Statistical analysis was performed using IBM's Statistical Page for Social Sciences (SPSS; version 19 for Apple Macintosh). Categorical data were analyzed using Pearson's chi‐squared test. Numerical data were analyzed using a one‐way ANOVA test and an independent sample *t*‐test. A *p*‐value of less than 0.05 was considered statistically significant. The protocol for this study was approved by the Human Research Ethics Committee at each participating hospital.

## 3 Results

From 1 January 2000 to 31 December 2023, 1353 patients underwent repair of a very large hiatus hernia. 733 (54.6%) were less than 70 years old, 451 (33.6%) were aged from 70 to 79 years, and 169 (12.6%) were 80 years or older. Baseline demographics and comorbidities are summarized in Table 1. Older and octogenarian patients were more likely to be female. Octogenarians had a lower body mass index and the highest rates of cardiovascular and respiratory disease.

**TABLE 1:** Baseline characteristics for all patients undergoing repair of a large hiatus hernia.

	< 70 years *n =* 733	70–79 years *n =* 451	≥ 80 years *n =* 169	*p*‐value
Demographics				
Age, years	59.7 (59.1–60.3)	74.5 (74.2–74.7)	83.9 (83.4–84.4)	< 0.001*
Female (%)	477 (65.1%)	322 (71.4%)	122 (72.2%)	0.036*
Height (m)	1.66 (1.65–1.67)	1.63 (1.62–1.64)	1.63 (1.61–1.65)	< 0.001*
Weight (kg)	84.12 (82.87–85.37)	75.73 (74.50–76.95)	69.84 (67.78–71.91)	< 0.001*
Body mass index, kg/m^2^	30.39 (29.96–30.81)	28.75 (28.29–29.21)	26.98 (26.20–27.76)	< 0.001*
Comorbidity				
Cardiovascular	55/424 (13.0%)	57/236 (24.2%)	20/65 (30.8%)	< 0.001*
Respiratory	172/676 (25.4%)	124/419 (29.6%)	53/152 (34.9%)	0.043*
Renal	9/373 (2.4%)	10/216 (4.6%)	3/58 (5.2%)	0.265
Diabetes	27/432 (6.3%)	27/240 (11.3%)	4/64 (6.3%)	0.062
Previous abdominal surgery	246/443 (55.5%)	154/244 (63.1%)	43/68 (63.2%)	0.112

*Note:* Data are mean (standard deviation/95% confidence intervals) or number (%).

The specific reasons for surgery are summarized in Table 2. As this information was available on the database for 787 of the overall cohort, patients where this information was not recorded were excluded from this aspect of the analysis. Younger patients (< 70 Years) were more likely to have undergone surgery primarily for reflux, whereas octogenarians were more likely to have undergone an urgent operation for gastric volvulus and/or obstruction. Other common reasons for surgery included volume regurgitation and bleeding/anemia. The frequencies of these presentations were similar across all groups. Patients under the age of 80 were more likely to have undergone preoperative endoscopy. Esophagitis at endoscopy was present more often in young and older patients.

**TABLE 2:** Preoperative details for all patients undergoing repair of a large hiatus hernia.

	< 70 years *n =* 459	70–79 years *n =* 259	≥ 80 years *n =* 69	*p*‐value
Reasons for surgery				
Heartburn	260 (56.6%)	115 (44.4%)	20 (29.0%)	< 0.001*
Regurgitation	157 (34.2%)	84 (32.4%)	15 (21.7%)	0.120
Bleeding/Anemia	130 (28.3%)	66 (25.5%)	18 (26.1%)	0.697
Epigastric pain	12 (3.1%)	11 (4.2%)	6 (8.7%)	0.076
Dysphagia	23 (5.0%)	25 (9.7%)	4 (5.8%)	0.053
Nausea and vomiting	6 (1.3%)	8 (3.1%)	7 (10.1%)	< 0.001*
Shortness of breath	17 (3.7%)	19 (7.3%)	12 (17.4%)	0.384
Cough	23 (5.0%)	14 (5.4%)	1 (1.4%)	0.380
Obstructing hernia	17 (3.7%)	19 (7.3%)	12 (17.4%)	< 0.001*
Volvulus	25 (5.4%)	17 (6.6%)	10 (14.5%)	0.019*
Preoperative investigations				
Endoscopy	666/733 (90.9%)	400/451 (88.7%)	140/169 (82.8%)	0.010*
Esophagitis	197/666 (29.6%)	111/400 (27.8%)	22/140 (15.7%)	0.004*
Barrett's esophagus	73/666 (11.0%)	46/400 (11.5%)	7/140 (5.0%)	0.078
Cameron's ulcers	25/666 (3.8%)	14/400 (3.5%)	5/140 (3.6%)	0.976
Contrast swallow	449/733 (61.3%)	274/451 (60.8%)	113/169 (66.9%)	0.344

*Note:* Data are mean (standard deviation/95% confidence intervals) or number (%). This table only includes patients where a reason for surgery was recorded in the database. When this data field was missing, patients were excluded. Patients may have multiple reasons for surgery.

Operative findings are summarized in Table 3. Large sliding hiatus hernias were less seen in the octogenarian groups, whereas total intrathoracic stomachs and very large mixed‐type hernias were more frequent in the older and octogenarian groups. A higher conversion rate to open surgery occurred in the octogenarian groups. Reasons for conversion included: inability to reduce the hiatus hernia contents laparoscopically, adhesions, bleeding, and esophageal injury.

**TABLE 3:** Operative details for all patients undergoing repair of a large hiatus hernia.

	< 70 years *n =* 733	70–79 years *n =* 451	≥ 80 years *n =* 169	*p*‐value
Hernia size				
> 50% intrathoracic	648/733 (88.4%)	359/451 (79.6%)	114/169 (67.5%)	< 0.001*
Total intrathoracic	85/733 (11.6%)	92/451 (20.4%)	55/169 (32.5%)	
Hernia type				
Sliding	209/706 (29.6%)	83/438 (18.9%)	12/160 (7.5%)	< 0.001*
Rolling/Paraesophageal	170/706 (24.1%)	88/438 (20.1%)	38/160 (23.8%)	
Mixed	327/706 (46.3%)	267/438 (61.0%)	110/160 (68.8%)	
Approach				
Laparoscopic	712/721 (98.8%)	430/438 (98.2%)	153/161 (95.0%)	0.002*
Conversion: Lap to open	8/721 (1.1%)	5/438 (1.1%)	8/161 (5.0%)	
Open	1/721 (0.1%)	3/438 (0.7%)	0/161 (0%)	
Reason for conversion				
Unable to reduce hernia sac	4/8 (50%)	2/5 (40%)	6/8 (75%)	0.725
Adhesions	2/8 (25%)	1/5 (20%)	2/8 (25%)	
Bleeding	1/8 (12.5%)	1/5 (20%)	0/8 (0%)	
Esophageal perforation	1/8 (12.5%)	1/5 (20%)	0/8 (0%)	
Hiatal repair				
Number of sutures	4.66 (4.54–4.78)	5.00 (4.85–5.16)	5.05 (4.79–5.32)	< 0.001*
Suture placement				
Anterior	7/717 (1.0%)	2/437 (0.5%)	2/168 (1.2%)	0.141
Anterior + posterior	241/717 (33.6%)	171/437 (39.1%)	70/168 (41.7%)	
Posterior	469/717 (65.4%)	264/437 (60.4%)	96/168 (57.1%)	
Mesh				
Mesh used	61/549 (11.1%)	43/350 (12.3%)	16/113 (14.2%)	0.629
Mesh type				
Absorbable	36/61 (59.0%)	25/43 (58.1%)	9/16 (56.3%)	0.980
Nonabsorbable	25/61 (41.0%)	18/43 (41.9%)	7/16 (43.8%)	
Fundoplication				
Wrap performed	724/730 (99.2%)	444/446 (99.6%)	166/167 (99.4%)	0.743
Fundoplication type				
90 anterior	109/724 (15.1%)	86/444 (19.4%)	44/166 (26.5%)	< 0.001*
180 anterior	492/724 (68.0%)	307/444 (69.1%)	108/166 (65.1%)	
270 posterior	36/724 (5.0%)	37/444 (8.3%)	5/166 (3.0%)	
360 nissen	87/724 (12.0%)	14/444 (3.2%)	9/166 (5.4%)	
Short gastric divided	61/724 (8.4%)	45/445 (10.1%)	13/165 (7.9%)	0.544
Bougie used	350/724 (48.3%)	185/442 (41.9%)	68/164 (41.5%)	0.055
Drain used	24/534 (4.5%)	23/353 (6.5%)	11/121 (9.1%)	0.109
Gastrostomy	1/674 (0.1%)	1/424 (0.2%)	2/150 (0.5%)	0.063
Collis‐gastroplasty	2/674 (0.3%)	1/424 (0.2%)	0/150 (0%)	0.798
Intraoperative complication	28/682 (3.9%)	16/429 (3.7%)	10/153 (6.7%)	0.321
Duration of surgery (minutes)	102.3 (99.1–105.4)	106.2 (101.8–110.6)	110.7 (104.1–117.4)	0.059

More hiatal repair sutures were used in the older and octogenarian groups and most sutures were placed posteriorly. There were no significant differences for mesh use. An anterior 180° fundoplication was the commonest fundoplication constructed. A Nissen 360° fundoplication was used more in younger patients, whereas an anterior 90° fundoplication was used more in octogenarians in the context of mechanical symptoms rather than reflux. A gastrostomy tube was used for gastropexy in 4 (0.3%) patients and a Collis gastroplasty was performed in 3 (0.2%) across all groups. There were no differences in intraoperative complications rates.

Postoperative outcomes are summarized in Table 4. Octogenarians stayed in hospital longer and were twice as likely to have a complication. Most complications were minor (Grade 2) cardiac or respiratory in nature. Major complications (Clavien–Dindo ≥ 3) were more common in the octogenarians. Early reoperation rates were low and similar for all groups and most commonly due to an early hiatus hernia recurrence (54.1%) or acute dysphagia (21.6%). Esophageal leak/perforation occurred in 5 cases. Mortality rates were low in all groups but higher in the octogenarian group at 30 days (0.3% vs. 0.4% vs. 1.8%) and 90 days (0.5% vs. 0.5% vs. 3.4%).

**TABLE 4:** Postoperative outcomes for all patients undergoing repair of a large hiatus hernia.

	< 70 years *n =* 733	70–79 years *n =* 451	≥ 80 years *n =* 169	*p*‐value
Length of stay (days)	2.69 (2.47–2.91)	3.19 (2.61–3.76)	4.62 (3.87–5.37)	< 0.001*
Postoperative complications				
All complications	68/713 (9.6%)	48/438 (11.1%)	31/160 (19.4%)	0.002*
Major (Clavien–Dindo ≥ 3)	30/712 (4.2%)	22/433 (5.1%)	13/160 (8.1%)	0.120
Complication details				
Grade 3	23/712 (3.2%)	14/433 (3.2%)	5/160 (3.1%)	
Grade 4	6/712 (0.8%)	6/433 (1.4%)	5/160 (3.1%)	
Grade 5	1/712 (0.1%)	2/433 (0.5%)	3/160 (1.9%)	
Early reoperation				
Early reoperation required	19/733 (2.6%)	13/451 (2.9)	4/169 (2.7%)	0.925
Reason				
Early recurrence	12/19 (63.2%)	7/14 (50%)	1/4 (25%)	
Dysphagia	2/19 (10.5%)	4/14 (28.6%)	2/4 (50%)	
Esophageal leak	2/19 (10.5%)	2/14 (14.3%)	1/4 (25%)	
Sepsis	1/19 (5.3%)	1/14 (7.1%)		
Bleeding	1/19 (5.3%)			
Gastric perforation	1/19 (5.3%)			
Death				
30‐day mortality	2/733 (0.3%)	2/451 (0.4%)	3/169 (1.8%)	0.048*
90‐day mortality	3/651 (0.5%)	2/405 (0.5%)	5/146 (3.4%)	0.001*
Late recurrence (within 12 m)				
Symptomatic recurrence	16/733 (2.2%)	9/450 (2.0%)	4/168 (2.1%)	0.956
Recurrence size				
Small	11/16 (68.8%)	7/9 (77.8%)	3/4 (75%)	0.937
Medium	3/16 (18.8%)	2/9 (22.2%)	1/4 (25%)	
Large	2/16 (12.5%)	0/9 (0%)	0/4 (0%)	
Reoperation rates	4/733 (0.5%)	2/451 (0.4%)	1/169 (0.6%)	0.962

*Note:* Data are mean (standard deviation/95% confidence intervals) or number (%).

To evaluate the impact of emergency presentations, subgroup analyses focusing separately on nonurgent elective and emergency procedures were performed (Table 5). For nonurgent elective surgery only patients, significant differences were no longer seen for conversion to open surgery, duration of surgery, major complication rates, and 30‐day mortality. However, even though 90‐day mortality rates were reduced, this remained greater in the octogenarian group (2.4%). Length of stay also remained longer and total complication rates were highest in the octogenarian group.

**TABLE 5:** Operative + postoperative outcomes subgroup analysis.

Nonurgent elective	< 70 years *n =* 699	70–79 Years *n =* 420	≥ 80 years *n =* 148	*p*‐value
Approach				
Laparoscopic	678/687 (98.7%)	400/407 (98.3%)	136/140 (97.1%)	
Conversion: Lap to open	8/687 (1.2%)	4/407 (1.0%)	4/140 (1.0%)	0.176
Open	1/687 (0.1%)	3/407 (0.7%)	0/140 (0%)	
Duration of surgery	101.7 (98.5–104.9)	104.4 (100.2–108.6)	109.3 (102.4–116.1)	0.130
Intraoperative complications	26/649 (4.0%)	15/399 (3.8%)	7/132 (5.3%)	0.734
Length of stay (Days)	2.63 (2.41–2.84)	3.06 (2.47–3.64)	4.19 (3.59–4.80)	< 0.001*
All complications	60/680 (8.8%)	39/408 (9.6%)	26/139 (14.2%)	0.002*
Major (Clavien–Dindo ≥ 3)	27/680 (4.0%)	16/404 (4.0%)	9/139 (6.5%)	0.386
Early reoperation	17/699 (2.4%)	10/420 (2.4%)	3/148 (2.0%)	0.957
Death				
30‐day mortality	2/699 (0.3%)	1/420 (0.2%)	1/148 (0.7%)	0.701
90‐day mortality	3/617 (0.5%)	1/375 (0.3%)	3/126 (2.4%)	0.027*

Urgent elective/emergency	< 70 years *n =* 34	70–79 years *n =* 31	≥ 80 years *n =* 21	*p*‐value
Approach				
Laparoscopic	34/34 (100%)	30/31 (96.8%)	17/21 (81.0%)	0.010*
Conversion: Lap to open	0/34 (0%)	1/31 (3.2%)	4/21 (19.0%)	
Duration of surgery	115.1 (98.5–131.7)	134.2 (102.5–165.9)	124.3 (95.4–153.2)	0.499
Intraoperative complications	2/33 (6.1%)	1/30 (3.3%)	3/21 (14.3%)	0.312
Length of stay (days)	3.91 (2.22–5.60)	5.07 (2.41–7.73)	7.60 (3.26–11.94)	0.167
All complications	8/33 (24.2%)	9/30 (30%)	5/21 (23.8%)	0.839
Major (Clavien–Dindo ≥ 3)	3/32 (9.4%)	6/29 (20.7%)	4/21 (19.0%)	0.433
Early reoperation	2/34 (5.9%)	3/31 (9.7%)	1/21 (4.8%)	0.752
Death				
30‐day mortality	0/34 (0%)	1/31 (3.2%)	2/21 (9.5%)	0.173
90‐day mortality	0/34 (0%)	1/30 (3.3%)	2/20 (10.0%)	0.160

Eighty‐six patients presented acutely with gastric volvulus or an obstructing hernia and were analyzed separately to the 1267 patients who presented for elective repair. Conversion to an open procedure was significantly higher in the octogenarian group. Intraoperative complications and length of stay were highest in the octogenarian group, but this did not achieve statistical significance. Complication and return to theater rates did not differ between groups. Although, 30‐day and 90‐day mortality rates were highest in the octogenarian group, this did not reach statistical significance.

Early symptomatic hernia recurrences detected by endoscopy or contrast swallow within 12 months of surgery did not differ between groups. Twenty‐one hernia recurrences were less than 2 cm in length and did not require repair. Eight recurrences were larger than 2 cm in length and were symptomatic. Seven of these 8 underwent reoperations during the 12 months after surgery.

Clinical symptom and satisfaction scores are summarized in Table 6. Preoperatively younger patients had higher heartburn and regurgitation severity scores. At median 12 (mean 9.68) months follow‐up, heartburn scores were significantly lower in all groups (*p* < 0.001 for all precomparison vs. postcomparison). Regurgitation severity scores also reduced significantly following repair across all age groups (*p* < 0.001 for all comparisons). Overall satisfaction after surgery was high in all groups, with scores in the octogenarian group significantly higher than the other age groups. At 12 months, most (92.2%–96.8%) patients indicated that they believed they had made the correct decision to undergo surgery.

**TABLE 6:** Symptom and satisfaction scores for all patients undergoing repair of a large hiatus hernia.

	< 70 years *n =* 733	70–79 years *n =* 451	≥ 80 years *n =* 169	*p*‐value
Heartburn (VAS 0–10)				
Preoperative score	5.92 (5.51–6.33)	4.76 (4.17–5.34)	4.72 (3.87–5.57)	< 0.001*
Postoperative score	1.18 (1.00–1.36)	0.77 (0.50–1.04)	0.89 (0.43–1.36)	0.085
Regurgitation (VAS 0–10)				
Preoperative score	6.26 (5.70–6.82)	5.18 (4.31–6.05)	4.33 (3.27–5.38)	0.004*
Postoperative score	1.01 (0.82–1.20)	0.64 (0.37–0.90)	1.14 (0.47–1.81)	0.208
Satisfaction score (out of 10)	8.81 (8.61–9.01)	8.77 (8.45–9.09)	9.39 (9.11–9.68)	0.030*
Would repeat surgery (yes/no)	371/395 (93.9%)	177/192 (92.2%)	90/93 (96.8%)	0.503

*Note:* Data are mean (standard deviation/95% confidence intervals) or number (%).

## 4 Discussion

Hiatal hernias are more common in older individuals for several reasons. As we age, the phrenoesophageal ligament becomes progressively laxer due to loss of elastic tissues from repetitive stress during swallowing [22]. Thoracic abnormalities which widen the anterior‐posterior diameter, such as kyphosis and pulmonary emphysema, are more common in the elderly and are associated with hiatal hernia formation [23]. Finally, repeated stress from pressure differentials between the abdomen and thorax overcomes the tensile strength of the hiatus contributing to both enlargement of hiatal hernias and recurrence after repair [24].

Large hiatus hernias are a significant source of morbidity and when they present emergently are associated with up to a 50% mortality rate [25]. Most published literature for large hiatal hernia repair defines “elderly or advanced age” as above 70 years [26, 27, 28]. However, given the increase in life expectancy, this needs to be reevaluated. Our study is one of the largest that compares outcomes following large hiatus hernias (≥ 50% intrathoracic stomach) repair in patients 80 years or over to a younger demographic.

In our study, octogenarians were more likely to undergo emergency surgery. They were also more likely to have a total intrathoracic stomach at surgery. This is consistent with other literature. Parker et al. found that octogenarians were more likely to present acutely (22.2%) and had a higher percentage of intrathoracic stomach (74.3%) [18]. Either octogenarians remain asymptomatic until they present acutely or there might be reluctance to refer for surgery or for surgeons to operate on octogenarians until they present acutely, even if symptomatic before this presentation. In contrast, patients under 70 years were more likely to undergo elective repair for heartburn. Younger patients appear more likely to be offered and pursue surgical treatment for reflux, whereas the selection threshold for older patients is likely to be higher, and ongoing treatment with medication for reflux symptoms is more likely to be encouraged.

Our study has shown higher rates of adverse outcomes in the octogenarian group, although the actual rates of complications and mortality appeared acceptably low, particularly when considered in the context of the octogenarians being more likely to present with an urgent or emergency issue with gastric volvulus. This higher rate of emergency procedures is also likely to contribute to the higher conversion from laparoscopic to open surgery in the octogenarians, and this conclusion is supported by the lack of difference after adjusting for only nonurgent elective cases.

Not unexpectedly, postoperatively octogenarians stayed in hospital 2 days longer, although major complications and early return to theater rates did not differ. Staerkle et al. found similar results from their propensity‐matched analysis of 360 octogenarians who underwent hiatus hernia repair. They included hernias of all sizes. General complications were higher for patients ≥ 80 years, but there were no significant differences in intraoperative and postoperative complications nor reoperations [9]. This also suggests that while recovery takes longer in the octogenarians, their perioperative major morbidity is low and similar to that of the younger population.

Our study also found that mortality rates in the elective setting in octogenarians was acceptably low (0.7%) and comparable to that of the younger population. DeMeester et al. reported an updated Markov analysis from patients with asymptomatic paraesophageal hernias. They found that between the ages of 40 and 90 years, elective laparoscopic hernia repair improves life expectancy over watchful waiting. The maximum benefit occurs for patients at 40 years, by adding an additional 2.6 years. In patients aged 80 years an older, elective surgery provides an additional 0.28 expected life years in females and 0.18 years in males [29].

We found that in the emergency setting a more than a 10‐fold increase in 30‐day mortality. All mortality needs to be considered within the context of urgent and emergency presentations where ongoing nonsurgical options are not going to be successful, and only surgery can resolve the mechanical issues of gastric obstruction from a very large hiatus hernia. Wilson et al. found similar mortality outcomes in a study which used a large‐scale nationwide database but with hiatus hernias of all sizes. They found that being an octogenarian was not independently predictive of mortality, but a nonelective operation was associated with a more than 4‐fold increase in mortality [8].

Our results differed somewhat to a recently published large study by Kumar et al. which compared octogenarians to “senior‐aged” patients (65–79 years) [7]. This study included both small and large hiatus hernias and found that octogenarians had a meaningfully increased risk of morbidity and mortality for both elective and emergency hiatal repair. Of note, octogenarians had a much higher rate of open surgery in the elective setting than in our study (4.58 vs. 1.0%). Their octogenarian 90‐day mortality rates were also higher, even in the elective setting (4.7 vs. 2.4%). This study differed from ours in that it did not separate hernias by size, it relied on retrospective analysis of hospital case records and administrative data rather than a prospective standardized data collection, and it provided no information about clinical symptom outcomes.

Our study demonstrated significant improvements in heartburn and regurgitation in all age groups, with almost all patients experiencing minimal to no symptoms 12 months after surgery. Octogenarians reported the highest satisfaction after surgery, and the majority believed they made the correct decision to have surgery. Khoma et al. also found that octogenarians also experienced a significant improvement in quality of life after surgery at early and late follow‐up [15].

There are some limitations to our current study. There are variable definitions for giant hiatal hernia in the literature, which can make comparison with other studies difficult. We chose to apply a conservative definition of > 50% of the stomach within the chest, whereas others have used a more liberal definition of > 30%. The > 50% definition used in our study ensured that all patients had a very large hiatus hernia. Despite the tighter definition, our cohort was still sufficiently large to answer the research question.

Most of our cohort (1267%–93.6%) underwent a planned elective operation. This reflects liberal selection for elective surgery. Only 86 (6.4%) were emergency presentations with acute gastric volvulus. Nasogastric tube decompression of the stomach was always attempted in these patients and usually successful, with most of these patients then scheduled for an elective procedure a few weeks later. However, even though emergency presentations were only 6.4% of the cohort, these 86 patients still represent a significant cohort, and the poorer outcomes in those patients, as well as less risk demonstrated for elective repair, encourages us to continue to recommend elective repair for most patients.

Unfortunately, missing data for the surgical indication meant that we were only able to determine and classify the reason for surgery in 787 (58.2%) patients. However, the cohort for whom this information was available was still large and the proportions of the different surgical indications are likely to still be accurate. Strengths of the study reported are the very large cohort of patients, inclusion of only patients with > 50% of their stomach in the chest, and exclusion of patients who underwent surgery before 2000 to ensure the surgical technique was consistent across the study period, and to avoid the impact of any learning curve on outcomes.

As life‐expectancy continues to rise, surgeons are seeing more patients above the age of 80 with very large hiatus hernias. Our study suggests that operating on these patients has an acceptable safety profile and provides excellent clinical outcomes, especially when surgery is undertaken electively. As such, age alone should not preclude patients being offered surgery for symptomatic very large hiatus hernias.

## Author Contributions

**Mathew A. Amprayil:** conceptualization, data curation, formal analysis, methodology, writing – original draft. **Tanya Irvine:** data curation, formal analysis, project administration, writing – review and editing. **Sarah K. Thompson:** methodology, project administration, supervision, writing – review and editing. **Tim Bright:** methodology, project administration, supervision, writing – review and editing. **David I. Watson:** conceptualization, data curation, formal analysis, methodology, project administration, resources, supervision, writing – review and editing.

## Conflicts of Interest

The authors declare no conflicts of interest.
